# Genetic architecture of primary sclerosing cholangitis: shared pathways with inflammatory bowel disease and gut–liver axis mediation

**DOI:** 10.1097/JS9.0000000000003594

**Published:** 2025-11-04

**Authors:** Yu Chen, Hui-Hong Zhang, Yu-Xuan Lu, Lin Lao, Shanying Liao, Jie Li, Shi-Xue Dai

**Affiliations:** a Clinical Medical School of Guangdong Provincial People’s Hospital, Southern Medical University, Guangzhou, Guangdong, China; b Department of Gastroenterology, Guangdong Provincial Geriatrics Institute, National Key Clinical Specialty, Guangdong Provincial People’s Hospital (Guangdong Academy of Medical Sciences), Southern Medical University, Guangzhou, Guangdong, China; c Department of Gastroenterology, Guangdong Provincial People’s Hospital (Guangdong Academy of Medical Sciences), Southern Medical University, Guangzhou, Guangdong, China; d Department of Critical Care Medicine, Guangdong Provincial Geriatrics Institute, National Key Clinical Specialty, Guangdong Provincial People’s Hospital (Guangdong Academy of Medical Sciences), Southern Medical University, Guangzhou, Guangdong, China; e Department of Intensive Care Medicine, Geriatric Center, National Regional Medical Center, Guangdong Provincial People’s Hospital Ganzhou Hospital, Ganzhou, Jiangxi, China; f Department of Gastroenterology, Geriatric Center, National Regional Medical Center, Guangdong Provincial People’s Hospital Ganzhou Hospital, Ganzhou, Jiangxi, China

**Keywords:** Genetic architecture, gut-liver axis, Genome-wide association study, Inflammatory bowel disease, Primary sclerosing cholangitis

## Abstract

**Background:**

Primary sclerosing cholangitis (PSC) is a chronic liver disease strongly linked to inflammatory bowel disease (IBD), yet its causal genetic drivers and the mechanisms underlying this comorbidity remain poorly understood. This study aimed to identify causal genes for PSC and elucidate the role of the gut-liver axis in its pathogenesis.

**Methods:**

We performed a multi-omics study integrating transcriptome-wide association studies (TWAS), summary Mendelian randomization (SMR), and colocalization using PSC GWAS data (2871 cases; 12 019 controls) and tissue-specific eQTL data. Bidirectional Mendelian randomization (MR) was employed to dissect causal relationships between identified genes, PSC, IBD, gut microbiota, and metabolites.

**Results:**

Seven genes were prioritized as potential causal targets for PSC: MMEL1, FUT2, PRKD2, C4A, HLA-DMA, VARS2, and RPL23AP1, with evidence supported by colocalization and expression in relevant immune and intestinal tissues. Bidirectional MR confirmed a causal link from IBD to PSC and identified shared genetic pathways. Crucially, MR analysis provided causal evidence for the role of specific gut microbiota in PSC risk, including increased risk with higher abundance of *Clostridium* and *Veillonella*. Mediation analyses further implicated FUT2 and HLA-DMA in modulating PSC risk via the gut microbiota, particularly through taxa such as *Clostridium, Butyrivibrio crossotus*, and *Rhodospirillaceae*.

**Conclusion:**

This study delineates PSC’s genetic architecture by identifying novel causal genes and confirms the gut-liver axis’s central role in its pathogenesis. We propose an integrated “dual-hit” model where host genetic susceptibility constitutes the “first hit” and subsequent gut dysbiosis acts as a “second hit.” These findings offer novel therapeutic targets and a mechanistic framework with potential implications for patient management in liver transplantation.

**HIGHLIGHTS**

- Identified seven potential causal genes (MMEL1, FUT2, PRKD2, C4A, HLA-DMA, RPL23AP1, and VARS2) associated with Primary Sclerosing Cholangitis (PSC).
- Revealed a bidirectional causal relationship between PSC and Inflammatory Bowel Disease (IBD), particularly with Ulcerative Colitis (UC).
- Demonstrated that four of the identified genes are related to the gut-liver axis, providing novel insights into PSC pathogenesis.
- Found that HLA-DMA and FUT2 influence PSC risk through modulation of gut microbiota composition.
- Utilized multi-omics genetic analysis, including transcriptome-wide association studies and Mendelian randomization, to explore PSC mechanisms.
- Highlighted the potential of the identified genes as future therapeutic targets for PSC management.

## Introduction

Primary sclerosing cholangitis (PSC) is a chronic liver disease characterized by bile duct inflammation and narrowing accompanied by bile duct fibrosis^[1]^. PSC can progress to severe liver fibrosis and cirrhosis, leading to bile duct narrowing, bile stasis, liver dysfunction, and death^[2]^. PSC is more commonly diagnosed among middle-aged men, and clinical research data suggest that the overall incidence rate in North America and European countries is 0.77 (0.45–1.09) per 100 000 person/year^[3]^. However, the overall prevalence and incidence rates showed an upward trend each year. The pathogenesis of PSC remains unconfirmed, and no treatment has been proven to effectively prolong the survival of patients with PSC, posing significant challenges in combating the disease.

In modern drug development, most candidate drugs fail during late-stage clinical trials, often because of poor prediction of their efficacy at early stages of target identification^[4,5]^. Recent studies have shown that drug targets with genetic support are more likely to be therapeutically valid^[4,6]^. However, utilizing genome-scale data from genome-wide association studies (GWAS) for drug target discovery in complex diseases still remains a challenging^[7]^. GWAS have been used to identify potential genetic risk factors for PSC and to discover potential drug targets. These GWAS have strongly linked thousands of genomic loci with complex traits. Despite such success, GWAS loci are often difficult to interpret; linkage disequilibrium (LD) often masks the causal variants driving the associations, and few of the effects of trait variation mediated by causal genes can be identified solely from GWAS data. This challenge of interpretation has prompted the development of various methods to prioritize causal genes at GWAS loci;^[8]^ however, there is still a lack of research using GWAS summary data to identify potential causal genes and drug targets for PSC with these new methods. Utilizing these methods can significantly enhance our understanding of the genetic basis of PSC and improve the development of targeted therapies.

New studies have outlined that a large percentage of people suffering from PSC also present with Inflammatory Bowel Disease (IBD), most commonly Ulcerative colitis (UC)^[2]^. This significant overlap makes PSC an ideal disease model to explore the gut-liver axis, which has inspired several studies regarding the pathogenesis of PSC, mainly on how the gut microbiome and its metabolites participate in PSC progression through the gut–liver axis^[9,10]^. Thus, these IBD-PSC relation studies and possible pathogenetic pathways have headed the dilemma, which came first, the chicken or the egg – that is, whether genetic susceptibility to IBD results in the occurrence of PSC through the gut-liver axis or vice versa. Although previous studies have elaborated on the robust genetic evidence that has validated a strong positive correlation between the genomic structures of PSC and IBD, research on the genetic interconnections between PSC and IBD is still in its early stages. To date, Mendelian randomization (MR) studies have shed light on a positive causal association between IBD, including UC, Crohn Disease (CD), and PSC in European cohorts. However, discussions that involve shared genetic susceptibilities, specter of reverse causality, and intricate pathogenic interplay between these conditions are obscure.

Our team identified the causal genes associated with PSC using comprehensive genetic and transcriptomic approaches, with further investigations into their causal effects on PSC using robust two-sample MR^[11]^ methods. In addition, considering the solid genetic correlation and comorbidity clinical evidence between PSC and IBD (especially UC)^[2,12]^, we further applied mediation MR analysis and colocalization tests to investigate whether these causal genes for PSC could also be shared causal loci for PSC and IBD or even further, key causal loci in the gut-liver axis that cause the occurrence of either disease from the other. Therefore, this study was designed not only to identify the specific genes that cause PSC but, more importantly, to understand how they contribute to the disease by mediating the pathological dialogue between the gut and the liver. Ultimately, our findings are intended to provide a genetic basis for stratifying patients at high risk of progression, identifying novel drug targets to prevent or delay the need for surgery, and potentially guiding personalized post-transplant management to reduce disease recurrence. In accordance with the Transparency In The reporting of Artificial Intelligence (TITAN) guideline^[13]^, we declare that no Artificial Intelligence (AI) tools were used in the research or development of this manuscript.

## Methods

### Study design

**Figure 1.**
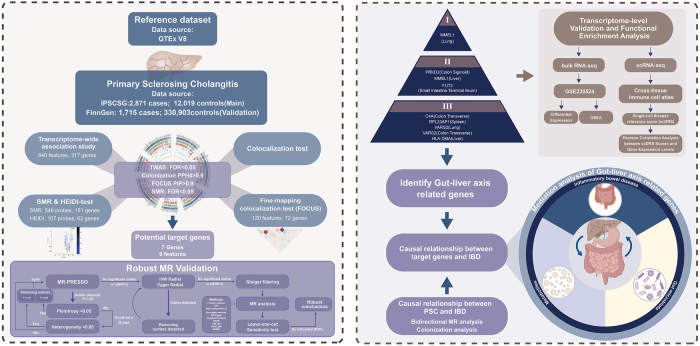
Graphical abstract, TWAS: Transcriptome-wide association study; SMR: Summary data based mendelian randomization.

We conducted a multi-omics integrative study to identify causal genetic drivers of PSC, combining transcriptome-wide association studies (TWAS), summary MR, Bayesian colocalization, and fine-mapping using GWAS data from IPSCSG and FinnGen. Tissue-specific eQTLs from GTEx v8 and immune cell single-cell RNA-seq data were integrated to prioritize genes. Bidirectional MR and mediation analyses evaluated causal relationships between PSC, IBD subtypes, gut microbiota, and metabolites. To ensure robustness, we implemented triangulation across methods (TWAS, SMR, colocalization) and datasets (IPSCSG, FinnGen), validated prioritized genes using scDRS scores in cross-tissue immune cells, and performed replication in independent cohorts. Statistical rigor was maintained via HEIDI tests (pleiotropy adjustment), FDR correction, and sensitivity analyses. This design ensured accuracy through multi-layered validation and minimized confounding bias. Detailed explanations of methodological approaches and threshold selections are provided in Supplemental Digital Content, Additional file 2: Extended Methods, available at: http://links.lww.com/JS9/F435. Overall, our research presents a significant contribution to the understanding of PSC by identifying potential therapeutic targets through an integrative biological approach (Fig. 1; Supplemental Digital Content, Additional file 3, available at: http://links.lww.com/JS9/F436).

### Data sources

We used the largest GWAS dataset for PSC from IPSCSG, with 2871 cases and 12 019 controls, covering 7.89 million SNPs^[14]^. The diagnoses followed strict clinical and histological criteria, excluding secondary sclerosing cholangitis. FinnGen r9 data (1491 cases, 301 383 controls) were used for validation^[15]^, with PSC cases identified via ICD-10. GWAS data for IBD, CD, and UC were sourced from the IEU database^[16]^, while gut microbiome data came from the MiBioGen consortium, covering 206 taxa from 18 340 participants^[17]^, and the Dutch Microbiome Project^[18]^. For microbiome-related metabolites, we used data on 40 metabolites from the IEU database, including pathways such as branched-chain amino acid and bile acid metabolism^[9,19]^. Data on specific metabolites were obtained from the studies by Borges *et al*^[20]^ and Shin *et al*^[21]^.

## Statistical analyses

### Identification of causal gene candidates

#### Heritability enrichment analysis

To pinpoint the most disease-relevant tissues and cell types for our investigation, we first performed heritability enrichment analysis using LDSC-SEG^[22]^. It estimates the genetic contribution of SNPs within the genomic range, and compared it with the genetic risk correlation of specific phenotypes, thereby guiding the selection of tissues for subsequent expression-based analyses.

#### Multi-method gene prioritization

To robustly identify genes whose expression levels are causally linked to PSC, we employed a multi-pronged strategy integrating four complementary methods:

Transcriptome-wide association study. We employed the FUSION method^[23]^ for transcriptome-wide association analysis. We systematically scanned for associations between genetically predicted gene expression in seven key tissues and PSC risk. Seven SNP weight sets from GTEx v8^[24]^ were obtained from the TWAS FUSION official website (http://gusevlab.org/projects/FUSION/#reference-functional-data).

Summary-data-based Mendelian Randomization. SMR analysis was used to test for a causal relationship between gene expression and PSC, while the integrated HEIDI test helped distinguish true causality from confounding due to linkage disequilibrium. A probe was deemed significant if it passed both SMR (FDR 0.05) thresholds. GTEx V8 eQTL data used for SMR analysis^[24]^ was obtained from the SMR official website.

Colocalization test. To test the hypothesis that a gene’s expression and PSC share the same underlying causal genetic variant, we performed Bayesian colocalization analysis for all significant TWAS genes. The “coloc” package in R implemented in FUSION was used to perform the analysis^[25]^.

Fine-mapping colocalization test. To further refine our causal gene list and pinpoint the most likely causal gene in regions with multiple signals, we used the FOCUS method^[26]^.

### Functional validation and profiling of candidates

#### Gene analysis and trait-score calculation using cross-tissue immune cell atlas data

Using MAGMA software^[27]^, we performed gene analysis on the GWAS data and quantified the total expression of presumed genes obtained from MAGMA results in each cell from the scRNA-seq data using scDRS (v1.0.2)^[28]^. scDRS scores were computed using the Cross-Tissue Immune Cell Atlas to link prioritized genes to disease-associated cell types.

#### Differential gene expression analysis and Gene Set Enrichment Analysis

The raw count data from GSE230524^[29]^ were transformed using log(x + 0.1) and normalized between samples using the normalizeBetweenArrays function from the limma R package^[30]^ before being used for subsequent analysis. Following data preparation, a *t*-test was employed to compare the expression levels of each target gene between the two sample groups to determine the statistical significance of any observed differences. Gene Set Enrichment Analysis (GSEA) was then conducted using the gseKEGG function from the clusterProfiler R package^[31]^.

### Mechanistic investigation of the gut-liver axis

#### Mendelian randomization analysis for validation and mediation analysis

To validate the causal effects of our candidate genes on PSC and to investigate the mechanistic pathways involving IBD and the microbiome, we conducted a series of two-sample MR analyses based on a robust analytical protocol^[32]^. After selecting instrumental variables (IVs) according to stringent criteria (see Supplemental Digital Content, Additional file 2: Extended Methods, available at: http://links.lww.com/JS9/F435), we primarily estimated causal effects using the fixed-effect inverse-variance weighted (IVW) method^[33]^ for exposures with multiple SNPs and the Wald ratio for those with a single SNP. To ensure the validity and robustness of these estimates, we implemented a comprehensive suite of quality control and sensitivity analyses. We systematically identified and removed outlier IVs using MR-PRESSO and RadialMR^[34]^, assessed directional pleiotropy with the MR-Egger intercept test, and evaluated instrument heterogeneity using Cochran’s Q statistic. Finally, to confirm our findings, the primary IVW results were corroborated using multiple secondary methods – including MR-Egger^[35]^, Constrained Maximum Likelihood and Model Averaging (CML-MA)^[33]^, and Bayesian Weighted Mendelian Randomization (BWMR)^[36]^ – to provide a comprehensive assessment and mitigate potential bias from horizontal pleiotropy.

To test the central hypothesis that our candidate genes influence PSC through the gut-liver axis, we performed a two-step MR analysis. This approach was conducted to investigate the potential pathways through which genes related to the gut–liver axis may influence PSC. Due to limited gut microbiota statistics, the associated SNP threshold was set at 1 × 10^−5^. A meta-analysis was conducted on shared microbiota statistics from the MibioGen and Dutch Microbiome Project for more accurate estimates.

## Results

### Heritability enrichment of PSC-relevant tissues and cell types

**Figure 2.**
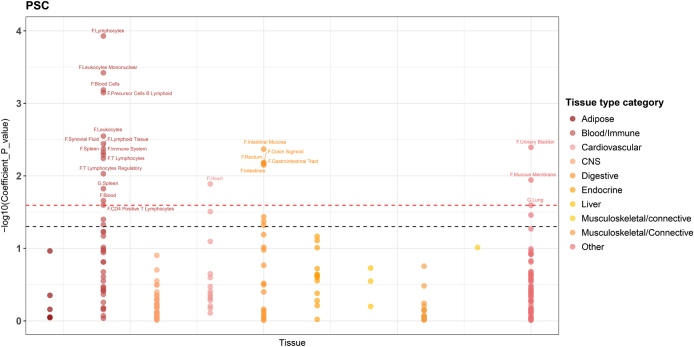
Results of the heritability enrichment analysis. Each dot in the plot represents a tissue or cell type from the GTEx dataset (N = 8,550) or Franke lab dataset (N = 37,427). The red line represents the cutoff value of FDR<0.25. The black line represents -log10(Coefficient P value) = 1.30 (i.E., P < 0.05).

To identify the biological context in which genetic risk variants are most likely to exert their functional effects, we performed a heritability enrichment analysis. Initial analysis of two gene expression datasets (GTEx, *N* = 8550 and Franke Lab, *N* = 37 427) revealed that PSC genetic heritability was mainly enriched in blood/immune and intestinal tissues (Fig. 2). Four immune-related tissues passed FDR < 5% correction: lymphocytes, leukocyte mononuclear cells, blood cells, and precursor B lymphoid cells, indicating a genetic link to immune cells in PSC development. No GTEx tissues passed FDR < 0.05, but by relaxing the threshold to FDR<0.25, the spleen and lungs were enriched (Supplemental Digital Content Table S1, available at: http://links.lww.com/JS9/F434). Following the TWAS guidelines^[8]^, seven tissues were selected for causal gene identification: spleen, lung, gut–liver axis tissues (liver, colon transverse, sigmoid colon, and small intestine terminal ileum), and whole blood as a reference. SNP weights were used to validate the causal genes across these tissues.

### Identification of the highest level of potential causal genes

**Figure 3.**
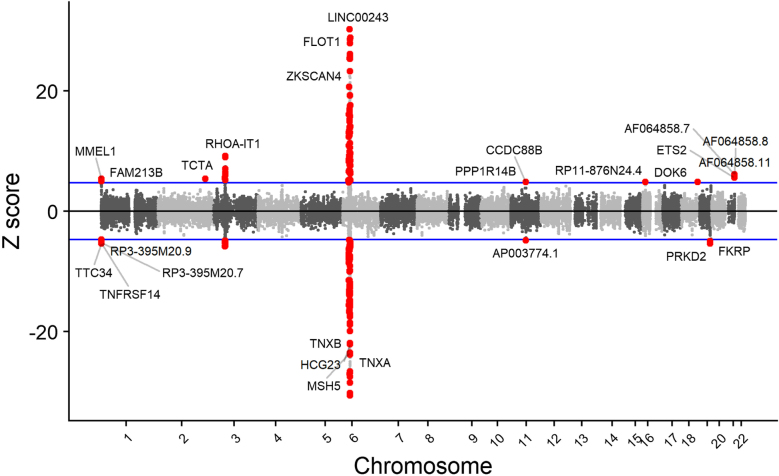
The correlation between gene expression and Primary Sclerosing Cholangitis from TWAS. (a) Manhattan plot at the gene level of TWAS results from the IPSCSG primary sclerosing cholangitis cohort. The X-axis represents the genomic position (based on NCBI Build 37), and the Y-axis represents the Z scores of TWAS. Manhattan-style plot of Z scores for each of the tested genes, across all autosomes and tested single nucleotide polymorphism weight sets. The blue lines indicate the transcriptome-wide significance threshold. All statistically significant genes are shown.

**Figure 4.**
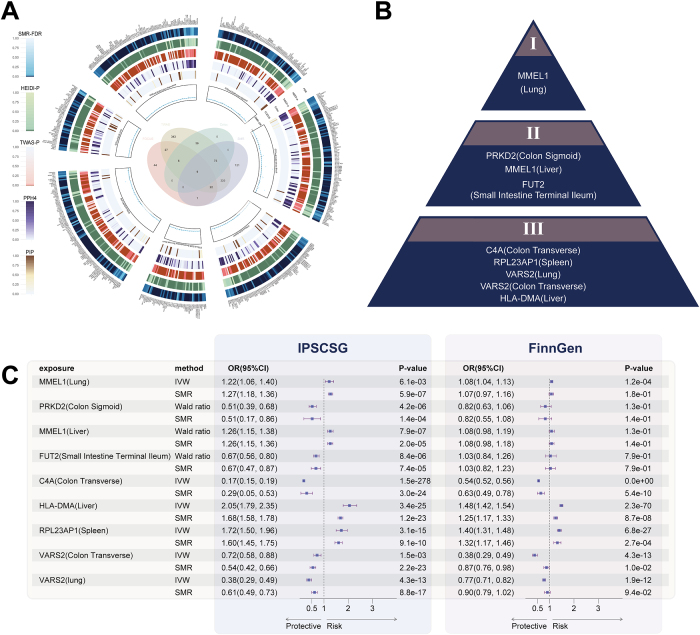
Analysis summary diagram: Identification of the highest level of potential causal genes. A. An annular heatmap showing the intersection and specific values of genes identified by SMR and the other three methods. Probes that were not found in the other methods are labeled with the least significant color in this method. From outer to inner, the blue circle shows the SMR-FDR values of all significant probes after FDR correction, the green circle shows the significance p-values of the corresponding HEIDI test, the red circle shows the p-values of each probe’s feature in the TWAS results, and the purple circle shows the values of PPH4 in the colocalization analysis. Dark purple represents features that passed the threshold of PPH4 > 0.9. The inner brown circle is used to show the PIP of each feature calculated by FOCUS, and dark brown represents features that passed the hypothesis threshold of PIP>0.9. B. All significant genes and their tissue sources in the first three levels of evidence. C. Forest plots showing the causal relationship and risk direction of all genes in the first three levels of evidence for PSC using multiple MR methods. This includes results from the IPSCSG database and the FinnGen database.

Heritability enrichment analysis of PSC revealed that genetic heritability was primarily concentrated in the blood/immune and intestinal tissues. Building on these findings, a transcriptome-wide association study (TWAS) was conducted, identifying 840 significant features across seven tissues, with RNF5 being the most notable. Conditional analysis revealed 77 jointly significant features that were co-expressed with PSC-related genes (Fig. 3). A colocalization test found 124 features from 57 genes sharing a causal variant with PSC (Supplemental Digital Content Tables S2–S4, available at: http://links.lww.com/JS9/F434). Fine-mapping identified 78 genes with strong causal relationships, including 45 with a PIP of 1 (Supplemental Digital Content Table S5, available at: http://links.lww.com/JS9/F434), and 64 were consistent with the TWAS results. Additionally, SMR analysis identified 236 probes, with 16 probes passing the HEIDI test and 546 probes under a more lenient FDR correction (Supplemental Digital Content Table S6, available at: http://links.lww.com/JS9/F434). Seven genes, namely MMEL1, FUT2, PRKD2, C4A, HLA-DMA, VARS2, and RPL23AP1, were identified as the most potential causal relationship value among all genes identified from seven tissues of GTEx v8 in the previous four methods (Fig. 4A).

To confirm the robustness of our identified targets, we used two-sample MR methods, carefully removed outliers, and validated the results using the FinnGen database. All targets showed positive results in these replicates (Fig. 4C). Our genetic analysis identified seven key genes associated with PSC, each influencing the disease risk differently (Table 1). For instance, MMEL1 in the lung tissue was linked to an increased PSC risk, while the C4A gene in the transverse colon significantly reduced the risk. HLA-DMA in the liver showed a strong association with higher PSC risk. Other genes, such as RPL23AP1, VARS2, PRKD2, and FUT2, also demonstrated significant associations, either increasing or decreasing PSC risk. All of these findings were statistically significant, highlighting the genetic influence on PSC susceptibility (Supplemental Digital Content Table S7, available at: http://links.lww.com/JS9/F434).

**Table 1:** Summary of prioritized causal genes for primary sclerosing cholangitis (PSC)

Gene symbol	Locus	Prioritized tissue	TWAS FDR	SMR FDR	HEIDI *P*-Value	Colocalization PPH4	FOCUS PIP	Type	Putative function
MMEL1	1p36.32	Lung	<0.001	<0.001	0.276	0.956	0.994	Protein coding	Membrane metallo-endopeptidase that degrades vaso- and immunomodulatory peptides, regulating autoimmune responses (NCBI Gene ID 79258).
		Liver	<0.001	<0.001	0.31	0.975	0.999		
FUT2	19q13.33	Small Intestine (Terminal Ileum)	<0.001	<0.001	0.151	0.991	0.962	Protein coding	α-1,2-fucosyltransferase synthesizing H-type 1 antigens required for intestinal mucosal barrier and gut-microbiota homeostasis (GeneCards FUT2).
PRKD2	19q13.42	Colon (Sigmoid)	<0.001	<0.001	0.761	0.921	0.999	Protein coding	Protein kinase D2; diacylglycerol-activated NF-κB signaling in colonic epithelium mediates inflammatory gene expression (GeneCards PRKD2)
C4A	6p21.33	Colon (Transverse)	<0.001	<0.001	<0.001	0.986	0.984	Protein coding	Complement component 4A, involved in innate immunity.
HLA-DMA	6p21.32	Liver	<0.001	<0.001	<0.001	1.000	1.000	Protein coding	MHC class II DM α-chain catalyzes CLIP release to enable antigen loading for CD4⁺ T-cell presentation (GeneCards HLA-DMA)
VARS2	6p21.33	Lung	<0.001	<0.001	<0.001	0.997	1.000	Protein coding	Encodes mitochondrial valyl-tRNA (Val-tRNA) synthetase, which is involved in mitochondrial translation (OMIM 612802)
		Colon (Transverse)	<0.001						
RPL23AP1	1p36.33	Spleen	<0.001	<0.001	<0.001	0.990	1.000	Transcribed unprocessed pseudogene	Ribosomal protein L23a processed pseudogene transcribed as non-coding RNA modulating ribosomal biogenesis (NCBI Gene ID 100507058)

CI, confidence interval; HEIDI, Heterogeneity in Dependent Instruments test; OR, odds ratio; PIP, Posterior Inclusion Probability in FOCUS fine-mapping; PPH4, Posterior Probability of Hypothesis 4 in colocalization analysis; PSC, primary sclerosing cholangitis; SMR, Summary-data-based Mendelian Randomization; TWAS, Transcriptome-Wide Association Study.

All data were meticulously extracted and cross-referenced from the provided supplementary tables. The most significant tissues for each gene are presented.

Putative Function: functional annotations derived from UniProt, NCBI Gene, OMIM and peer-reviewed literature; full citations are listed in the supplementary references.

However, when we validated these findings using the FinnGen database, FUT2, PRKD2, and MMEL1 from the liver did not show significant results. Based on these outcomes, we graded the identified targets according to their levels of evidence (Supplemental Digital Content Table S8, available at: http://links.lww.com/JS9/F434, Fig. 4B). The highest level included MMEL1 in the lungs, which showed significant results in all four GTEx-based analysis methods, passed the HEIDI test, and had significant MR results in the FinnGen data. The next level comprised MMEL1 in the liver, FUT2, and PRKD2, which showed strong results and passed the HEIDI test but lacked causal validation in FinnGen. The levels of C4A, RPL23AP1, VARS2, and HLA-DMA, which showed significant associations, were validated in FinnGen but did not pass the HEIDI test, indicating potential pleiotropy. Despite this, they are still considered potential causal genes because the potential pleiotropy affects only the MR assumption and not the robustness of other evidence.

### Validation of genetic risk genes through transcriptome analysis

**Figure 5.**
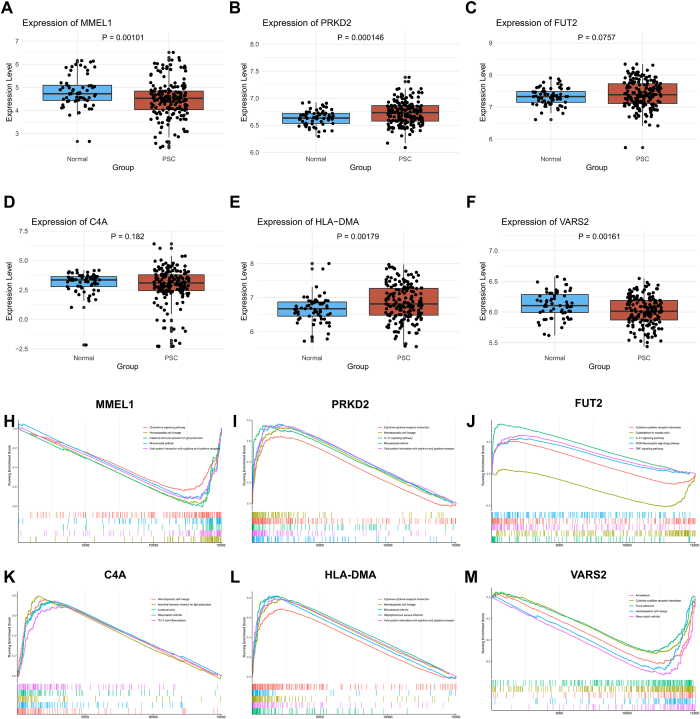
Differential expression and pathway analysis of genetic risk genes in PSC patients. (A-F) Box plots showing the expression levels of six genetic risk genes between normal individuals (blue) and PSC patients (red). Each dot represents an individual sample. (G-M) GSEA plots depicting the enrichment of immune-related pathways for the corresponding genes. These results highlight the significant expression differences and associated pathways of these genes, suggesting their involvement in the immune response of PSC patients.

To validate and investigate the potential roles of genetic risk genes in PSC inflammation, we analyzed their expression differences in the inflamed intestines of normal individuals and patients with PSC using bulk RNA-seq data. Patients were grouped based on gene expression levels and GSEA was conducted to explore the associated pathways. Significant differences were observed for MMEL1 (*P* = 0.001), PRKD2 (*P* = 0.0001), HLA-DMA (*P* = 1.7 × 10^−3^), and VARS2 (*P* = 1.6 × 10^−3^) between PSC patients and controls (*P* < 0.008; Fig.5 A-5F). GSEA results (Supplemental Digital Content Table S9, available at: http://links.lww.com/JS9/F434) showed that MMEL1 was downregulated in immune pathways such as the hematopoietic cell lineage and intestinal immune network. PRKD2 is upregulated in inflammation-related pathways, including cytokine interactions and IL-17 signaling. FUT2 was upregulated in pathways involving apoptosis and immune responses, such as IL-17 and TNF signaling. C4A is upregulated in infection defense and bone remodeling pathways, such as hematopoietic cell lineage and osteoclast differentiation. HLA-DMA was upregulated in immune regulation pathways and VARS2 was downregulated, suggesting an inhibitory role in immune responses (Fig. 5H–M). These findings highlight the complex and interconnected roles of different genes in the immune response of PSC patients.

**Figure 6.**
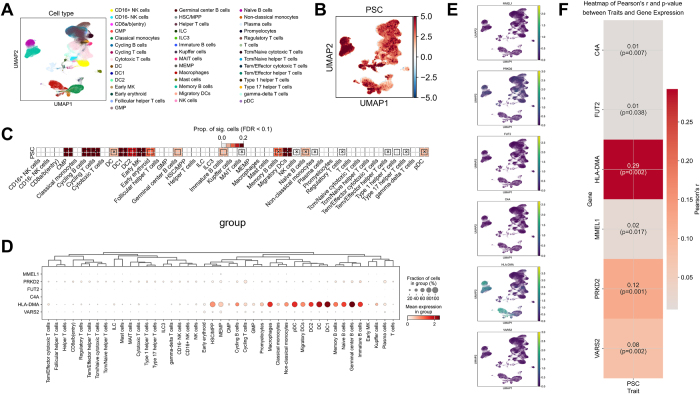
Single-cell RNA-seq analysis of potential PSC targets across various immune cell types. A. UMAP visualization of major immune cell types identified in the dataset. B. Overview of scDRS results overlayed on the UMAP, displaying computed scDRS disease scores. This visualization highlights the strong enrichment of specific cell types in PSC pathology. C. Heatmap showing the proportion of cells significantly associated with each cell-type trait pair. The colors represent the proportion of cells with significant associations, and squares indicate significant associations between cell types and traits (FDR < 0.1 across all combinations of cell types and traits). Cross symbols denote significant heterogeneity in the association with traits among individual cells within a specific cell type. D. Dot plot illustrating the fraction and average expression levels of PSC-associated genesacross different immune cell types. Larger dot size represents a higher fraction of cells expressing the gene, and the color intensity indicates higher average expression levels. E. UMAP plots showing the expression distribution of selected PSC target genes (C4A, FUT2, HLA-DMA, MARE1, PRKD2, VARS2) across different cell types. F. Heatmap of Pearson’s correlation coefficients (r) and p-values between PSC targets’ expression levels and scDRS scores.

### Validation of PSC targets through cross-tissue immune cell expression analysis

To further validate the relevance of the targets to the disease and confirm the accuracy of the targets through triangulation, we used cross-tissue immune cells to verify whether the expression levels of these targets were related to the scDRS scores of the disease, that is, to verify whether these targets were highly expressed in disease-related cells. We first used MAGMA to identify PSC GWAS summary statistical data from IPSCSG and mapped the obtained disease-related gene sets to single-cell datasets using the scDRS tool (Fig. 6A). Using annotations from the original literature, we assessed the correlation between cell types and PSC at the group level. At the cell group level, we found various cell types to be correlated with PSC. These include CMP, cyclin B cells, Cycling T cells, DC, DC1, DC2, early MK, early erythroid, germinal center B cells, Immature B cells, Memory B cells, MAIT cells, Migratory DCs, Naive B cells, non-classical monocytes, Regulatory T cells, Tem/effector helper T cells, type 1 helper T cells, type 17 helper T cells, and pDC. Among them, CMP, cyclin B cells, cyclin T cells, DC1, DC2, Early MK, Memory B cells, Migratory DCs (Fig. 6C), and pDC showed the strongest correlation with PSC (Supplemental Digital Content Table S10, available at: http://links.lww.com/JS9/F434).

Simultaneously, we visualized the expression levels of potential PSC targets in various cell types in this scRNA-seq dataset (Fig. 6D and E) and evaluated the correlation between the expression levels of these genes and the scDRS scores using Pearson correlation analysis. The results showed that all targets were significantly correlated with the PSC scDRS scores, indicating that these targets were significantly enriched in genetically related cells (Fig. 6F). Among them, HLA-DMA (*r* = 0.29, *P* = 0.002) and PRKD2 (*r* = 0.12, *P* = 0.001) particularly showed a strong correlation with scDRS scores (*r* > 0.1). Notably, HLA-DMA was highly expressed in disease-related cell types, and cell types with high expression levels of HLA-DMA almost coincided with the significant results obtained from scDRS.

### Identification of shared pathogenic targets between PSC and IBD and causal genes related to the gut-liver axis

**Figure 7.**
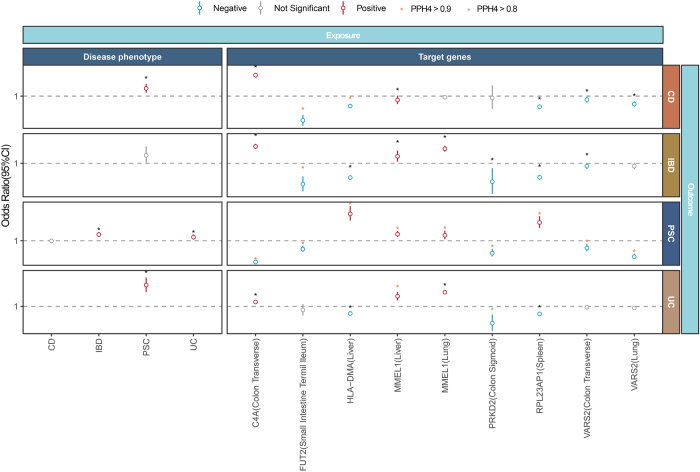
Bidirectional MR analysis (IVW or Wald ratio method) results showing the relationship between Primary Sclerosing Cholangitis and Inflammatory Bowel Disease (including Crohn’s disease and Ulcerative Colitis) and colocalization analysis results using 7 candidate gene targets (9 features in total) as exposures and PSC and three phenotypes of IBD as outcomes.

To identify common therapeutic targets and potential intermediate relationships between PSC and IBD, a bidirectional MR analysis of the relationship between PSC and IBD was first performed after removing outliers, and a significant causal relationship between IBD-PSC (P_IVW_ = 4.1 × 10^−8^), UC-PSC (P_IVW_ = 3.9 × 10^−3^), PSC-CD (P_IVW_ = 4.1 × 10^−4^), and PSC-UC (P_IVW_ = 1.1 × 10^−2^) was confirmed by the egger-intercept test for pleiotropy and heterogeneity tests. The positive causal relationships were consistent with previous studies, which observed significant causal relationships between IBD and UC with PSC but not observe a causal relationship between CD and PSC^[37]^. In reverse MR analysis, primary sclerosing cholangitis (PSC) demonstrated causal associations with UC and CD. Seven candidate causal genes showed relationships with IBD phenotypes under multiple correction thresholds (P < 1.9 × 10^−3^), with nearly all genes associating with at least one IBD subtype (Fig. 7). Colocalization analysis (PPH4 > 0.9) strengthened evidence for four gut-liver axis genes simultaneously satisfying MR and colocalization criteria: MMEL1 (liver)-UC (P_Wald_ = 6.1 × 10^−0^; PPH4 = 0.91), FUT2 (ileum)-CD (P_IVW_ = 1.9 × 10^−10^; PPH4 = 0.99) and IBD (P_IVW_ = 4.12 × 10^−0^; PPH4 = 0.98), and HLA-DMA (liver)-IBD (P_IVW_ = 1.95 × 10^−33^; PPH4 = 0.96). PRKD2-UC approached significance (PIVW = 3.0 × 10^−0^; PPH4 = 0.88), suggesting potential IBD relevance. Notably, MHC region exclusion maintained robust PSC-IBD associations (P_IVW_ = 7.69 × 10^−10^ for IBD, 2.74 × 10^−12^ for CD, 1.51 × 10^−10^ for UC). Given complex PSC-IBD interactions and reverse causality post-MHC exclusion, mediation analysis was omitted to avoid unreliable conclusions. These findings identify shared causal genes between PSC and IBD, with additional method details in Supplemental Digital Content Tables S11–S13, available at: http://links.lww.com/JS9/F434.

### Examining the mechanisms underlying the influence of gut-liver axis-related genes on primary sclerosing cholangitis

**Figure 8.**
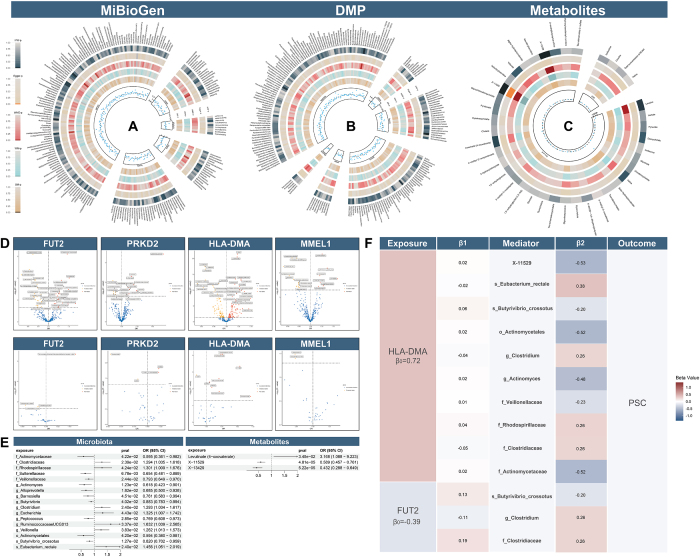
Examining the Mechanisms of Gut-liver Axis Gene Influence on Primary Sclerosing Cholangitis and Causal Relationships with Gut Microbiota and Metabolites. A. Results of five MR analysis methods on gut microbiota abundance and PSC in the MiBioGen database. B. Results of five MR analysis methods on gut microbiota abundance and PSC in the DMP database. C. Results of five MR analysis methods on gut microbiota-related metabolites in PSC. IVW: Inverse-variance Weighted, Egger: MR-Egger, WME: Weighted Medium, WM: Weighted Mode, SM: Simple Mode. D. Volcano plot showing the effects of four gut-liver axis-related targets estimated by the IVW or Wald ratio method on the gut microbiota and associated metabolites. MMEL1 and PRKD2 did not show significant results after FDR correction, so only uncorrected p-values are shown. E. Mendelian randomization (MR) positive results of gut microbiota with PSC after meta-analysis of the same microbiota in two databases using the IVW method, as well as positive results of gut microbiota-related metabolites with PSC. F. Illustration of the target-mediator-outcome relationship established in MR. Β0 represents the total effect from exposure to outcome, β1 represents the effect from exposure to mediator, and β2 represents the effect from mediator to outcome.

To investigate gut-liver axis gene influences on primary sclerosing cholangitis (PSC), we performed MR analyses on four target genes, gut microbiota, and microbiota-related metabolites. MR results identified specific gut microbiota taxa causally linked to PSC risk (Fig. 8A–C). Meta-analysis of two microbiome datasets (Supplemental Digital Content Tables S16–S17, available at: http://links.lww.com/JS9/F434, Figure 8E) revealed increased PSC risk associated with elevated abundance of *family Clostridiaceae* (P_IVW_ = 0.024), *species Eubacterium* rectale (P_IVW_ = 0.024, OR = 1.46), *genus Clostridium* (P_IVW_ = 0.024, OR = 1.29), genus *Ruminococcaceae* UCG013 (P_IVW_ = 0.033, OR = 1.63), *genus Veillonella* (P_IVW_ = 0.038, OR = 1.26), *family Rhodospirillaceae* (P_IVW_ = 0.042, OR = 1.30), and *genus Escherichia* (P_IVW_ = 0.044, OR = 1.32). Conversely, reduced risk was observed with *family Sutterellaceae* (P_IVW_ = 0.007, OR = 0.65), *family Veillonellaceae* (P_IVW_ = 0.024, OR = 0.79), *family Actinomycetaceae* (P_IVW_ = 0.042, OR = 0.59), *genus Actinomyces* (P_IVW_ = 0.012, OR = 0.61), *genus Alloprevotella* (P_IVW_ = 0.018, OR = 0.68), *genus Peptococcus* (P_IVW_ = 0.088, OR = 0.028), *genus Butyrivibrio* (P_IVW_ = 0.040, OR = 0.88), *genus Barnesiella* (P_IVW_ = 0.045, OR = 0.76), order Actinomycetales (P_IVW_ = 0.041, OR = 0.59), and *species Butyrivibrio crossotus* (P_IVW_ = 0.013, OR = 0.82).

For metabolites (Supplemental Digital Content Tables S18–S19, available at: http://links.lww.com/JS9/F434), X-11529 (P_IVW_ = 4.81 × 10^−5^, OR = 0.58) and X-13429 (P_IVW_ = 5.22 × 10^−5^, OR = 0.43) were inversely associated with PSC risk (FDR < 0.05), whereas levulinate (4-oxovalerate) increased risk (P_IVW_ = 0.034, OR = 3.16) (Fig. 8B and E). All associations passed pleiotropy and heterogeneity tests.

Among gut-liver axis genes, FUT2 (terminal ileum) and HLA-DMA (liver) showed significant microbiota/metabolite associations post-FDR correction (FDR<0.05), including FUT2’s positive correlation with branched-chain amino acids (Fig. 8D). MMEL1 (liver) and PRKD2 (sigmoid colon) exhibited no significant links. Three mediation pathways including HLA-DMA→*Rhodospirillaceae*→PSC; FUT2→*Clostridium*→PSC; FUT2→*Butyrivibrio crossotus*→PSC, met criteria but failed Sobel testing (Fig. 8F; Supplemental Digital Content Table S21, available at: http://links.lww.com/JS9/F434). Full sensitivity analyses and MR method results are detailed in Supplemental Digital Content Tables S14–S20. available at: http://links.lww.com/JS9/F434.

## Discussion

Our research provides a comprehensive analysis of the complex genetic background and pathogenesis of PSC through systematic multi omics causal inference. It not only identifies new risk and protective gene loci, but also confirms at the causal level that the imbalance of the gut liver axis is the core link mediating host genetic susceptibility and ultimately leading to the pathological process of PSC. These findings are highly consistent with previous clinical studies and, in conjunction with a groundbreaking animal model study on the FUT2 gene, have jointly constructed an integrated PSC “dual-hit” model with a novel pathophysiological basis^[38,39]^.

First, our study identified host genetic factors associated with PSC risk. We confirmed that the FUT2 gene, located on chromosome 19q13, has a significant protective effect on PSC. FUT2 regulates the unfolded protein response to protect intestinal stem cells from inflammatory damage, thereby playing a role in the occurrence and development of IBD. Loss-of-function mutations of FUT2 are associated with PSC, Crohn’s disease, and biliary tract injury^[40]^, and its disruption in animal models can lead to liver disease. The functional state of FUT2 is determined by a specific SNP (rs601338), a finding that is consistent with the conclusion that the non-secretor state of FUT2 is an important genetic risk factor for PSC. In the long-term exploration of the mechanism of action of FUT2, a pivotal animal model study by Maroni *et al* provided a key anatomical and pathophysiological explanation for the mechanism of action of FUT2^[39]^. They used a FUT2-knockout mouse model (simulating the human “non-secretor” risk state), they found that approximately half of the mice did not develop primary cholangitis but instead exhibited a significant congenital vascular defect: portosystemic shunting (PSS). This anatomical anomaly allows portal blood to bypass the liver’s first-pass effect and enter the systemic circulation directly, providing a physical basis for understanding how the FUT2 gene influences diseases via the gut-liver axis. Furthermore, our analysis identified other key genes, notably MMEL1 gene located on chromosome 1p36, which was associated with a significantly increased the risk of PSC. Previous research has linked MMEL1 to the susceptibility to primary biliary cirrhosis^[41,42]^, as well as to various autoimmune diseases including rheumatoid arthritis^[43]^, psoriasis^[44]^, UC^[45]^, and type 1 diabetes^[46]^. This finding suggests that therapeutic strategies targeting MMEL1 must carefully consider the complex risk-benefit profile.

This study’s core contribution is to provide causal evidence that changes in specific gut microbiota are pathogenic factors for PSC, which is highly consistent with the aforementioned “dual-hit” model. We utilized the most comprehensive gut microbiome GWAS data and related metabolomics data from two databases to explore the role of the gut liver axis in explaining the relationship between gut microbiota and PSC pathogenesis, and to link them to potential therapeutic targets. Our MR analysis showed that an increase in the abundance of pro-inflammatory bacterial communities such as *Veillonella, Escherichia*, and *Clostridium*, as well as a decrease in the abundance of butyrate-producing bacterium *Eubacterium rectale*, were all pathogenic factors for PSC. These findings provide strong empirical support for the dual-hit model proposed by Maroni *et al*^[39]^. Congenital portal shunt caused by FUT2 gene deletion is the “first-hit,” creating an anatomical vulnerability; the harmful bacteria and their products identified in the gut constitute the “second hit.” At the same time, experimental evidence shows that mice carrying portal shunts rapidly develop fatal liver damage after exposure to toxic bile acids from human sources, which strongly demonstrates the anatomical “highway” of portal shunts, greatly amplifying the pathological impact of harmful substances from the gut on the liver. Under this mechanism, the causal pathway revealed by our mediation analysis of HLA-DMA, which reduces PSC risk by increasing the abundance of beneficial *Rhodospirillaceae*, has deeper pathological significance, that is, by regulating the gut microbiota to directly weaken the intensity of the “second hit.”

This new model, which integrates genetics, anatomy, and microbiology provides a novel perspective for the clinical practice of PSC, especially for optimizing the prognosis of liver transplantation. While orthotopic liver transplantation (OLT) is the only curative option for end-stage PSC patients^[47]^, postoperative bile duct stenosis (BS) and disease recurrence (recPSC) seriously affect long-term patient survival^[48]^. Previous studies have confirmed that persistent postoperative intestinal inflammation, especially active colitis, is a key risk factor for these complications^[49]^. The study by Maroni *et al* suggests that some PSC patients (especially FUT2 non-secretor individuals) may have subclinical portal shunts. This implies that even after liver transplantation, inflammatory mediators, and harmful microbial metabolites from the intestine may continue to assault the transplanted liver through residual or newly formed collateral circulation, driving disease recurrence. This model also explains why traditional immunosuppressants such as tacrolimus can partially alleviate symptoms by inhibiting T-cell activation, but still cannot fundamentally resolve the underlying imbalance of the gut–liver axis.

Despite its strengths, our study has several limitations that warrant consideration. First, the presence of confounding factors and reverse causality made it challenging to validate some conclusions through randomized controlled trials. Second, the reliance on a single dataset (GTEx v8) may limit the generalizability of our results, underscoring the necessity of using more diverse genomic data sources in future research. Furthermore, our microbiome analysis lacks GWAS sequencing data on the biliary microbiota, which may play a crucial role in the pathogenesis of PSC. At the same time, the genetic data we studied mainly came from European populations, and the generalizability of our research conclusions in other ethnicities needs further verification. Future studies should aim to establish animal models to further explore the mechanisms of the gut-biliary-liver axis, which could unveil new therapeutic targets.

More importantly, based on our existing research findings and previous studies on clinical treatment strategies for PSC, we propose a dual strategy of “gut microbiota intervention combined with anti-inflammatory therapy” aimed at addressing both the “source” and “pathway” during the perioperative period for PSC patients. One key component of this approach is the reshaping of the gut microbiota. Through interventions like fecal microbiota transplantation or precise probiotic/prebiotic administration, the goal is to correct the patient’s gut microbiota imbalance, reduce the abundance of pathogenic bacteria (e.g., *Clostridium*), and restore intestinal barrier function. This is expected to fundamentally weaken the intensity of the “dual-hit.” The second component involves targeted anti-inflammatory therapy. Existing anti-inflammatory and immunosuppressive regimens (e.g., α4β7 integrin inhibitors) should be continued or optimized to control the downstream immune responses triggered by intestinal inflammation. We believe this dual-pronged approach can more effectively block the pathological cycle driving transplanted liver injury, thereby significantly reducing the risk of postoperative bile duct stenosis and disease recurrence. Future clinical trials are needed to validate the effectiveness of this integrated treatment strategy and to explore the clinical value of FUT2 genotyping and portal shunt screening for PSC patients.

## Conclusion

In summary, we identified seven potential causal genes for PSC, four of which are associated with the gut–liver axis, providing new insights into PSC pathogenesis. Our bidirectional MR analysis deepened our understanding of the relationship between PSC and IBD and the role of gut microbiota in PSC. These findings, considering the lack of effective PSC treatments, could guide the development of targeted therapies and help identify novel drug targets within the gut-liver axis. Future studies should focus on validating these targets in a clinical setting and exploring the potential of these genes as predictive biomarkers and therapeutic targets for disease management and treatment.

## Data Availability

The datasets analyzed in this study are publicly accessible from the following sources: Primary Sclerosing Cholangitis (PSC) GWAS summary data were obtained from the NHGRI-EBI GWAS Catalog, available at https://www.ebi.ac.uk/gwas/; supplementary validation data for PSC were sourced from FinnGen r9, accessible at https://www.finngen.fi/fi; GWAS summary data for Inflammatory Bowel Disease, Crohn’s Disease, and Ulcerative Colitis were sourced from the IEU database at https://gwas.mrcieu.ac.uk/; the summary GWAS data for the gut microbiota were procured from the MiBioGen consortium and the Dutch microbiota Project, available at https://mibiogen.gcc.rug.nl/ and the IEU database (https://gwas.mrcieu.ac.uk/). GWAS data for 40 microbiota-related metabolites, including those from the pathways of valine, leucine, and isoleucine metabolism; bile acid metabolism; glycolysis, gluconeogenesis, and pyruvate metabolism, were acquired from the IEU database at https://gwas.mrcieu.ac.uk/. SMR analysis utilized GTEx v8 eQTL summarized data, available from the SMR official website at https://yanglab.westlake.edu.cn/software/smr; gene expression data, including 153 tissues or cell types from the Franke lab dataset and 52 tissues and cell types from the GTEx dataset can be downloaded from https://console.cloud.google.com/storage/browser/broad-alkesgroup-public-requester-pays; the original contributions presented, including All IVs used in the MR analysis, along with F-statistic for each instrumental variable, the scDRS score of each individual cell in the scRNA-seq data, and other supplementary figures in the study are included in Supplementary Materials or available at figshare (https://figshare.com/articles/dataset/Other_Supplementary_Materials/25150673); Further inquiries can be directed to the corresponding authors.

## Research registration unique identifying number (UIN)

Since this is a retrospective study using publicly available genomic datasets, a prospective clinical trial registration is not applicable. The study utilized de-identified data from public repositories, including GWAS databases and GTEx project, which do not require clinical trial registration.
